# Agenesis of isthmus of thyroid gland in adult human cadavers: a case series

**DOI:** 10.1186/1757-1626-2-6640

**Published:** 2009-04-20

**Authors:** Daksha Dixit, MB Shilpa, MP Harsh, MV Ravishankar

**Affiliations:** 1 Department of Anatomy, KLE University's Jawaharlal Nehru Medical College, Belgaum, Karnataka, India

## Abstract

The thyroid gland, a highly vascular endocrine gland, is composed of two lateral lobes connected by a narrow median isthmus thus giving an 'H' shaped appearance to the gland. A wide range of morphological variations and developmental anomalies of the thyroid gland have been reported in the literature. In our study, on the morphometric features of the thyroid gland it was found that, 6 out of 41 thyroid glands that were dissected, showed an absence of the isthmus. The respective lateral lobes were positioned independently on either side of the trachea. The incidence of agenesis of isthmus, along with the developmental and clinical significance are discussed herein under.

## Introduction

The thyroid gland, brownish-red and highly vascular endocrine gland, is placed anteriorly in the neck, extending from the fifth cervical to the first thoracic vertebrae. It is ensheathed by the pre-tracheal layer of deep cervical fascia. The gland is composed of two lateral lobes connected by a narrow median isthmus. The normal size of each lobe of the thyroid gland has been described to be 5 cm long, its greatest transverse and anteroposterior extent being 3 cm and 2 cm respectively. The isthmus measures about 1.25 cm transversely as well as vertically and is usually placed anterior to the second and third tracheal cartilages [1]. The anomalies of the development of the thyroid gland distort the morphology of the gland, and may cause clinical functional disorders and various thyroid illnesses [2]. Incidence of agenesis of the thyroid isthmus has been reported to vary from 5% to 10% by Pastor et al and from 8% to 10% by Marshall [3,4]. Ranade et al in their study on anatomical variations of the thyroid gland reported a 33% incidence of agenesis of the isthmus [5]. The knowledge of various developmental anomalies of the gland and variations in neurovascular relations will help the surgeon in better planning of a safe and effective surgery.

## Case presentation

**Figure 1:**
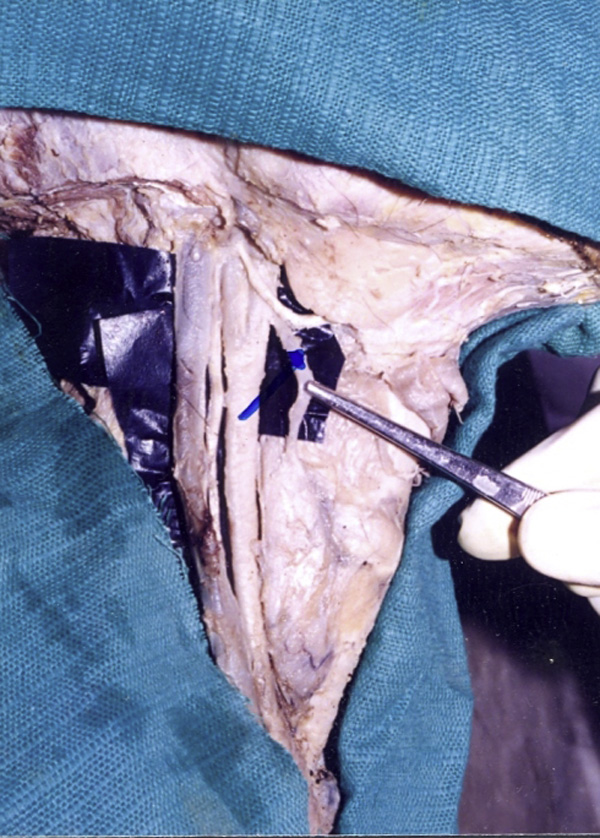
**Showing the superior thyroid artery**. The superior thyroid artery is seen originating from the external carotid artery. The internal jugular vein and Vagus nerve are also seen alongside the common carotid artery.

**Figure 2:**
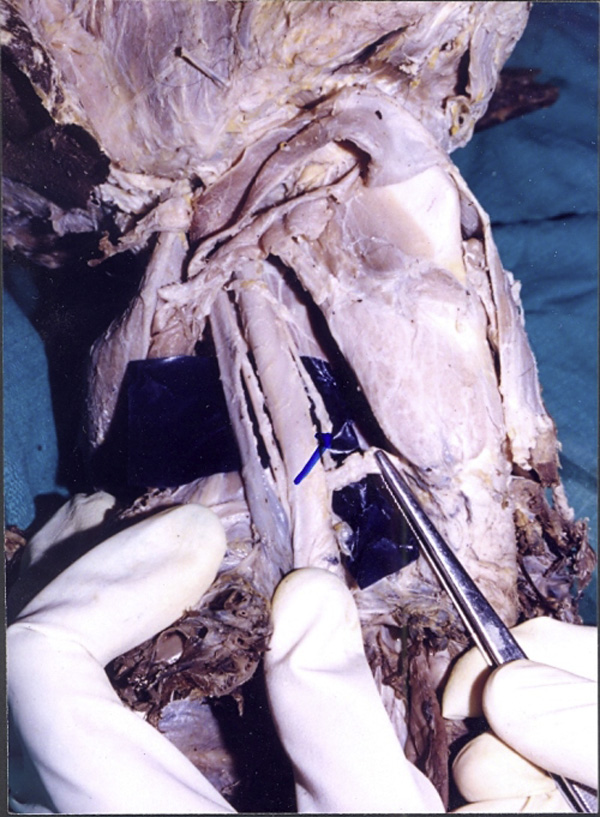
**Showing the recurrent laryngeal nerve and inferior thyroid artery**. We can see the inferior thyroid artery supplying the lower pole of the thyroid gland. Also seen is the recurrent laryngeal nerve running deep to the inferior thyroid artery.

The aims and objectives of our study were to study the morphometric features of the thyroid lobes and isthmus, and also to note the variations in neurovascular relations. Our study included dissection of the thyroid gland in 41 adult human cadavers all aged between 37 and 64 years, out of which 37 were male and 4 were female cadavers. Various parameters like length of the lateral lobes, height of isthmus, presence of pyramidal lobe and levator thyroidae glandulae, origin of the arteries supplying, and termination of the veins draining the thyroid gland were recorded as follows:

The average length of the right lobe of thyroid gland was 5.29 cm and that of the left lobe was 4.95 cm. The average height of the isthmus was 2.25 cm. The pyramidal lobe and levator thyroidae glandulae were both present in 3 cases (7.31%). In 38 cases (92.68%) the superior thyroid artery originated from the external carotid artery whereas the inferior thyroid artery was a branch of the thyrocervical trunk in all the cadavers (Figure 1). The thyroidae ima artery was present in 1 cadaver (2.43%). The superior thyroid vein drained into the internal jugular vein in 31 out of 41 cases (75.6%) whereas in all the 41 cadavers the middle thyroid vein drained into the internal jugular vein. The inferior thyroid veins drained into the left brachiocephalic vein in 40 cases (97.56%). The recurrent laryngeal nerve, on the right side, was superficial to the inferior thyroid artery in 14 cases (34.14%), deep to it in 26 cases (63.41%) and in 1 case it travelled through the branches of the artery (Figure 2). On the left side, it was superficial to the artery in 5 cadavers (12.19%) and deep to it in 36 cases (87.8%).

**Figure 3:**
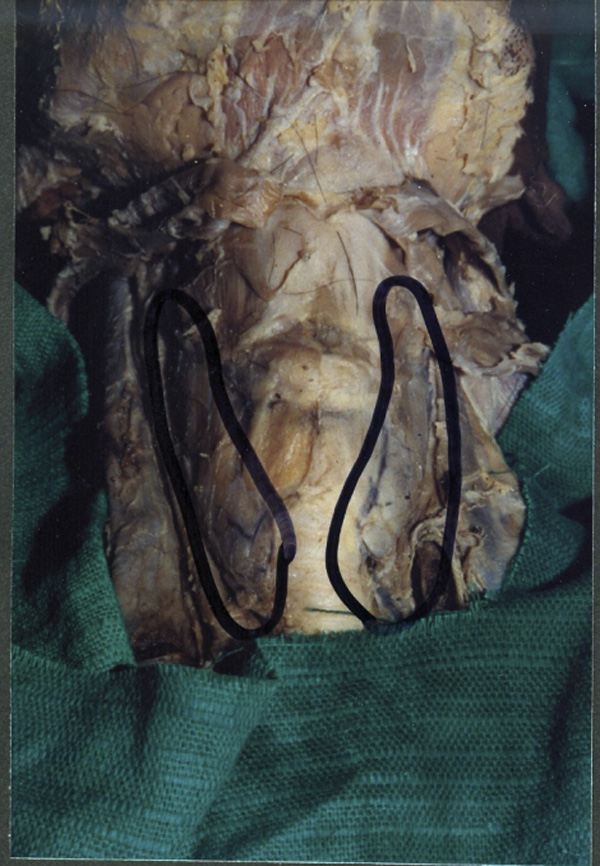
**Showing a case of agenesis of isthmus of thyroid gland**. We can see the two lateral lobes lying independently on either side of the trachea connected by a thin layer of pre-tracheal fascia.

During midline dissection of the neck 6 out of the 41 cadavers dissected showed no glandular tissue in the region of the isthmus of thyroid gland. Grossly, only the pre-tracheal fascia connecting the right and left lobes of the thyroid gland was observed (Figure 3).

The average lengths of the right and left lobes in these 6 cadavers were 5.01 cm and 4.71 cm respectively. The male to female ratio of incidence of agenesis of isthmus was 5:1 in our study. There were no significant variations in neurovascular relations and no ectopic thyroid tissues were found.

## Discussion

Agenesis of the thyroid isthmus is the complete and congenital absence of the thyroid isthmus as is defined by Pastor et al [3]. In their study, they had reported agenesis of isthmus of thyroid gland with enlarged lobes in a Caucasian cadaver. According to Gruber (quoted by Testus and Latarjet) the incidence of agenesis of isthmus is about 5% [6]. Marshall documented the variations in the gross structure of the thyroid gland in 60 children, varying in age from a few weeks to 10 years and the absence of the isthmus was reported to be 10% in this group [4]. Ranade et al reported absence of isthmus in 35 out of 105 cases (33%), of which 8 were female cadavers [5]. According to the study by Braun et al, the isthmus was missing in 4 cases of the 58 cadavers they studied [7]. Won and Chung have reported that in 3% of the cases studied, the isthmus was absent and the lateral lobes of the thyroid were separated [8]. The incidence in Northwest Indians is reported to be 7.9% in gross specimens [9]. In our study the incidence was a little higher at 14.6%.

**Figure 4:**
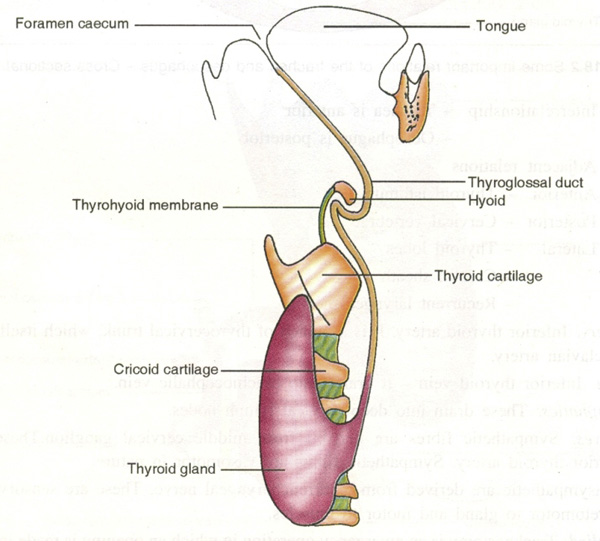
**Showing the development of thyroid gland**. The thyroid gland begins to develop as a median thickening of endoderm on the floor of the pharynx between the first and second pharyngeal pouches. This area later invaginates to form the median diverticulum, which appears in the later half of the fourth week. This thyroid diverticulum grows further, becoming a solid cellular cord called the thyroglossal duct. The duct grows caudally and bifurcates to give rise to the thyroid lobes and the isthmus.

Absence of isthmus is quite rare in humans [2]. The agenesis of isthmus can be explained as an anomaly of embryological development. The adult thyroid gland has two types of endocrine cells, follicular and parafollicular cells or 'C' cells, which are derived from two different embryological cell families. The follicular cells come from the endodermic cells of the primitive pharynx and the parafollicular cells come from the neural crest [10]. The thyroid gland begins to develop as a median thickening of endoderm on the floor of the pharynx between the first and second pharyngeal pouches. This area later invaginates to form the median diverticulum, which appears in the later half of the fourth week. This thyroid diverticulum grows in allometric proliferation, becoming a solid cellular cord called the thyroglossal duct. The duct grows caudally and bifurcates to give rise to the thyroid lobes and the isthmus (Figure 4). At the same time that its caudal growth is taking place, the cephalic end of the thyroglossal duct degenerates [11].

A high division of the thyroglossal duct can generate two independent thyroid lobes with the absence of isthmus. The absence of the isthmus can be associated with other types of dysorganogenesis, such as the absence of a lobe or the presence of ectopic thyroid tissue [12].

Clinically, the diagnosis of agenesis of the isthmus can be done with scintigraphy, which can also be performed with an overload of TSH. The diagnosis can also be done with the aid of ultrasonography, computerized tomography (C.T.), magnetic resonance imaging (M.R.I.) or during a surgical procedure. In asymptomatic patients with nodular goitres fine-needle aspiration biopsies and eventually immunohistochemistry tests are useful to support the medical decision but when agenesis is present the importance of pre-operative differentiation between benign and malignant lesions is critical, considering the surgical procedure and the possibility of impairment of the thyroid function [13]. When an image of the absence of isthmus is observed, a differential diagnosis against autonomous thyroid nodule, thyroiditis, primary carcinoma, neoplastic metastasis and infiltrative diseases such as amyloidosis should be considered [3].

## Conclusion

Agenesis of isthmus of thyroid gland is rare in humans, the incidence varying from 5% to 10%. In our study the incidence was 14.6% with a male to female ratio of 5:1. This agenesis can be explained as an anomaly of embryological development i.e. a high division of the thyroglossal duct giving rise to two independent thyroid lobes with absence of isthmus. Agenesis of isthmus can be associated with other types of dysorganogenesis, such as the absence of a lobe or the presence of ectopic thyroid tissue and hence in clinical practice when such a condition is diagnosed, it is necessary to perform a differential diagnosis against other pathologies such as autonomous thyroid nodule, thyroiditis, etc. The surgeon planning a thyroidectomy must be prepared to find variations like ectopic thyroid nodules around the normally-located thyroid gland. Proper identification of vessels is very important in order to avoid major complications. Hence a thorough knowledge of the thyroid anatomy and its associated anatomical variations is very much essential, so that these anomalies are not overlooked in the differential diagnosis.

## List of abbreviations

CT: computerized tomography; MRI: Magnetic resonance imaging.

## Consent

Written informed consent was obtained from the relatives of all the subjects for publication of this case series and accompanying images. Copies of the written consents are available for review by the Editor-in-Chief of this journal.

## Competing interests

The authors declare that they have no competing interests.

## Authors' contribution

DD conceived the study, obtained written consent and wrote the case series. SMB and HMP did the literature search and MVR helped to draft the manuscript. All authors read and approved the final manuscript.
